# Navigating interprofessional collaboration in diabetes care: A qualitative study of early-career health professionals in malaysian primary care clinics

**DOI:** 10.1371/journal.pone.0335192

**Published:** 2025-10-28

**Authors:** Kelly Sze Fang Num, Azimatun Noor Aizuddin, Syahnaz Mohd Hashim, Mohd Shahrir Mohamed Said

**Affiliations:** 1 Department of Public Health Medicine, Faculty of Medicine, Universiti Kebangsaan Malaysia, Kuala Lumpur, Malaysia; 2 International Centre for Casemix and Clinical Coding, Hospital Canselor Tuanku Muhriz, Kuala Lumpur, Malaysia; 3 Department of Family Medicine, Faculty of Medicine, Universiti Kebangsaan Malaysia, Kuala Lumpur, Malaysia; 4 Department of Internal Medicine, Universiti Kebangsaan Malaysia Medical Center (UKMMC), Kuala Lumpur, Malaysia; Queensland University of Technology - QUT: Queensland University of Technology, AUSTRALIA

## Abstract

**Introduction:**

Interprofessional collaborative care (IPC) is essential for effective healthcare delivery, particularly in managing chronic conditions such as diabetes in primary care settings. However, early-career health professionals (ECHPs) often encounter significant challenges when establishing effective IPC, given its inherent complexity. This study explores how ECHPs in primary care clinics navigate and engage in IPC for diabetes management.

**Methods:**

A qualitative study was conducted from 1^st^ December 2021–1^st^ October 2022 at two Malaysian primary care clinics (urban and suburban). Seven ECHPs meeting predefined criteria (6 months to 5 years’ experience, no postgraduate degree) were purposively sampled and interviewed until data saturation. In-depth semi-structured interviews (face-to-face or virtually via Zoom), conducted in either English or Malay, were audio- or video-recorded and transcribed verbatim. Data were analyzed using Braun and Clarke’s reflexive thematic analysis with constant comparison to ensure rigor.

**Results:**

Seven main themes emerged regarding how ECHPs in primary care clinics navigate and engage in interprofessional collaborative practices: (1) Initiating and continuing dialogue, (2) Creating cohesiveness, (3) Effective ways of communication, (4) Having own personal values, (5) Willing to work synergistically, (6) Learning from each other, and (7) Embracing diversities and resolving conflict. These themes represent interrelated components that ECHPs had adopted to effectively engage in interprofessional collaborative practices.

**Conclusion:**

IPC in diabetes management is a complex system requiring ECHPs to employ interrelated components for effective engagement. ECHPs overcame hierarchical barriers through proactive dialogue, reflecting a shift toward egalitarian teamwork. Digital platforms aided coordination, though face-to-face interactions were preferred for complex cases and direct communication. Team cohesion was strengthened through shared leadership, conflict resolution, and interprofessional learning, enabling ECHPs to adapt and contribute confidently. Educational institutions should integrate emotional intelligence, negotiation skills and digital ethics into IPC curricula. Healthcare organizations must reinforce collaborative practices through policies, mentorship and structured training to bridge theory-practice gaps. Future research should explore informal socialization, peer coaching and long-term digital communication impacts to strengthen IPC support for ECHPs.

## Introduction

Interprofessional collaborative care (IPC) has been recognized as a cornerstone of comprehensive healthcare, especially in managing diabetes, a chronic condition that requires long-term multidisciplinary care. Effective diabetes management relies on IPC, where doctors, pharmacists, nurses, diabetes educators, and allied health professionals combine [1–3] expertise to deliver holistic patient care [1,2,4–7]. As the demand for patient care grows increasingly complex, health professionals (HPs) must possess not only technical expertise but also the ability to collaborate effectively within teams [1,8]. Moreover, the dynamic nature of primary care settings requires that early-career HPs (ECHPs) rapidly develop clinical proficiency alongside the interpersonal and collaborative skills essential for IPC [9,10]. The IPC process is inherently complex, which requires ECHPs to navigate professional hierarchies, manage and coordinate patient care within multidisciplinary teams, and build confidence in their practice simultaneously [11–13].

As the healthcare landscape continues to evolve, there is a growing emphasis on integrating IPC into health professional education. Many Malaysian medical and healthcare university programs have begun actively adopting the World Health Organization (WHO) Interprofessional Collaborative Practice Framework into undergraduate curricula, aiming to equip HPs with the collaborative skills needed across disciplines [14–16]. While it is hoped that ECHPs will apply these interprofessional skills in clinical practice, the extent to which this IPC integration effectively translates into clinical practice remains uncertain [15,17].

While IPC is increasingly integrated into health professional education, ECHPs often struggle to translate theoretical teamwork knowledge into real-world clinical practice [10,15,17–19] This gap raises concerns about how well academic IPC training prepares ECHPs for the complexities of collaborative diabetes management in primary care. [15–17,20–22].

Effective collaboration abilities among ECHPs and other HPs is crucial for maintaining continuity of care, ensuring medication adherence, and delivering patient education. However, ECHPs often encounter challenges in navigating collaborative practices as they transition from internships to the independent clinical workforce [9,20]. Research on how HPs engage in IPC during the early stages of their career remains underexplored, particularly in primary care clinics, where they encounter difficulties in establishing interprofessional collaborative care practices (IPCPs), when managing chronic conditions such as diabetes [3,9,20]. These challenges arise from multiple factors: professional factors, such as varying levels of clinical expertise and confidence in decision-making [23]; organizational factors, including hierarchical structures, heavy workload demands, and resource constraints [24] and interpersonal factors, such as communication barriers, professional identity conflicts and team dynamics [25]. During university learning or internships, some ECHPs may have gained exposure to IPC principles or practices, while others might have experienced only task delegation within multidisciplinary teams, without fully understanding or engaging in interdependent collaborative relationships [26].

This study explores how ECHPs in primary care clinics navigate and engage in IPC when managing diabetes. By exploring their lived experiences, this research addresses the gap in understanding how ECHPs navigate IPC during early stages of their careers, particularly in the primary care context. The research findings can inform both educators and primary healthcare clinic leaders in refining educational curricula and organizational strategies to enhance IPCPs among early-career HPs, ultimately leading to more effective team-based diabetes management and improved patient care in primary care.

## Methods

### Research design, study setting, participants, and sampling

This is a qualitative study, conducted between 1^st^ December 2021, and 1^st^ October 2022 at two primary care clinics: a) Klinik Primer Hospital Canselor Tuanku Muhriz, an urban public university clinic, and b) Klinik Kesihatan Hulu Langat Batu 14, a public clinic in a suburban community. Both clinics serve diverse patient populations, offering a valuable opportunity to explore how ECHPs navigate IPC in contrasting clinical settings. Detailed descriptions of these clinics’ services have been published previously [9].

Participants were purposively sampled according to the following early-career health professional criteria: [1] full-time Malaysian HPs; [2] having six months to five years of experience managing and consulting diabetes care; [3] not having obtained any postgraduate degree. These criteria captured ECHPs’ perspectives during formative practice years. The sampling approach ensured representation across professional roles, experience levels, and practice settings to obtain diverse viewpoints. Semi-structured interviews began with the first participant and continued until data saturation was reached at the seventh interview, when no new themes or insights emerged.

### Ethical approval

Ethical approval was obtained from the Universiti Kebangsaan Malaysia Medical Research Ethics Board (JEP-2021–639) and the Ethics and Medical Research Committee, Ministry of Health Malaysia [NMRR-21-1646-60768 (IIR)].

### Ethics statement

This study was conducted in accordance with the Declaration of Helsinki. An information sheet (see S1 File) detailing the research purposes and data collection processes was provided and explained to the participants. Written informed consent (see S2 File) to participate in this study was obtained from all participants before commencing any data collection activities.

### Data collection

Data were collected through in-depth interviews (IDIs) conducted in accordance with the Consolidated Criteria for Reporting Qualitative Research (COREQ) guidelines [27]. These interviews explored participants’ detailed experiences and thought processes regarding IPCPs in diabetes care. All seven participants were interviewed once by the first author, a PhD candidate with 11 years of experience as a dietetics lecturer.

Prior to each interview, participants were comprehensively briefed about the research purposes and data collection processes, after which they provided written informed consent. Interviews were conducted using a semi-structured interview topic guide (see S3 File) in private, comfortable, and convenient settings that accommodated participants’ schedules. Interview sessions were held either face-to-face at the clinic premises or virtually via Zoom, with all participants opting to keep their video cameras activated. This allowed for observation of non-verbal cues, including facial expressions and body language, in both modalities.

Across all interview sessions in both modalities, the first author maintained uniform interview and probing techniques. Participants could choose to conduct the interview in either English or Malay, based on their preference. Each interview session lasted 45–60 minutes and was audio- or video-recorded with participants’ consent. Participants’ confidentiality was protected through secure storage protocols.

### Data analysis

The data were analyzed using Braun and Clarke’s (2022), six-step reflexive thematic analysis [28–30], strengthened by the constant comparison method [31–34]. Qualitative researchers have employed deductive, inductive, or summative approaches for qualitative analysis [35–42]. In our study, an inductive thematic analysis was adopted, which followed a data-driven coding process which allowed patterns to emerge organically without imposing pre-existing theoretical constructs [28–30,43,44].

The first author performed all transcriptions (English and Malay language) using Microsoft Word’s transcription tool, followed by repeated immersive readings of the transcripts [45,46]. Analytical memos were documented throughout this stage to capture emerging patterns that were aligned with research objectives [47,48]. After this immersion phase, the transcripts were systematically coded in NVivo 10.0 using a primarily inductive, line-by-line coding approach consistent with Braun and Clarke’s (2022) reflexive approach, while remaining sensitive to relevant theoretical models and prior work on IPC [28,29]. This approach allowed our interpretation to contextualize participant narratives with broader scholarly understanding. For examples of initial codes included *“Referral new or old patients,” “Paying initial visits to other units,” and “Review current patient”*. Significant data segments were identified and cross-compared across transcripts to ensure analytical consistency [45,46,49]. Malay-language verbatim quotes underwent the identical analytical approach to maintain methodological rigor.

All initial codes from both language datasets were then exported to Microsoft Excel for thematic development. The spreadsheet format enhanced visual organization of codes which systematically facilitating their grouping into broader themes [50,51]. According to Alyaqoub et al.’s model, themes can be extracted deductively from theory or inductively from raw data [35]. Our analysis was guided by such flexibility, drawing on qualitative coding best practices [28,36–38,41,43]. For example, the sub-themes “Making the first move and bridging the gap,” “Making referrals,” “Discussing patient continuity of care” emerged from the clustered codes and broader theme generated as “Initiating and continuing dialogues”, reflecting how participants begun proactively engaged in collaborative care. Our sub-themes were developed using granular approach to preserve the nuanced distinctions that emerged from participants’ narratives. This detailed delineation aligns with contemporary thematic analysis principles that emphasize preserving semantic richness and avoiding premature conceptual compression that could obscure meaningful differences in participants’ experiences [29,30,49,52,53]. Furthermore, our granular structure reflects the current literature of interprofessional collaboration being complex, multidimensional process involving distinct and interrelated components [10,11,54,55].

The research team then refined themes through iterative review cycles. This process involved revisiting original transcripts to verify thematic coherence and data completeness. Final themes were explicitly defined and refined. A final results report based on the established themes and subthemes was written.

The analysis employed constant comparative techniques throughout the research process, continuously analyzing new data against existing codes and themes. This approach ensured the analytical framework comprehensively represented the full range of participants’ experiences while systematically identifying and reconciling any contradictions or variations within the dataset [28,30,33].

Additionally, Malay-language verbatim quotes in the study were coded first in their original language to preserve linguistic and cultural authenticity [56–58]. Selected Malay-language quotes underwent rigorous translation into English through back-translation and forward-translation to ensure semantic equivalence [56,57,59,60] This dual-translation approach minimized meaning distortion while retaining response integrity [57,60]. Bilingual research team members validated translations for accuracy and contextual appropriateness [57,59,61]. In the final report results, all quotes underwent *“light tidying up”* to enhance readability by clarifying local colloquialisms and addressing ethical considerations while carefully preserving their genuineness [62–64].

### Researcher reflexivity and trustworthiness

The first author’s position as a dietetics lecturer with 11 years of academic experience required caution regarding potential influences on the study processes. While the author’s background facilitated rapport-building with participants, the author was aware of the risk of encouraging socially desirable responses about collaborative practices [65,66]. This concern was addressed by maintaining an ongoing reflexive journal to identify assumptions and potential biases that might influence data interpretation.

Before each interview, the first author shared her professional background with participants while emphasizing the study’s objective was to explore collaborative care experiences, including collaborative failures, rather than to evaluate clinical competency. Participants were assured of confidentiality and reminded that there were no ‘right’ or ‘wrong’ answers to encourage open discussions. Neutral probing techniques were adopted to minimize interviewer influencing on participants’ narratives [67,68].

Triangulation was embedded at multiple stages to enhance credibility. Participants were purposefully selected from diverse professional roles, contributing varied perspectives on IPCPs. Bilingual data sources were analyzed in their original language to minimize meaning distortion. Regular research team meetings systematically monitored data collection and analysis progress, facilitated discussion of analytical memos, and resolved emerging coding discrepancies. The research team conducted regular peer debriefings to examine data interpretations and identify potential researcher assumptions beyond those captured in analytical memos.

## Results

### Participants

Seven participants in this study (Table 1) were predominantly female and represented various healthcare roles, with the majority being medical officers alongside one diabetes educator, one pharmacist, and one occupational therapist. Most participants had three to four years of work experience, while two participants had five years, and one had one to two years. Regarding educational background, five participants held bachelor’s degrees, and two had diplomas.

**Table 1:** Participants’ characteristics data (N = 7).

Participant ID	Age	Gender	Education status	Occupation	Work experiences in year
HP1	29	Female	Bachelor	Medical officer	4
HP2	31	Male	Bachelor	Medical officer	5
HP3	38	Female	Diploma	Diabetes educator	5
HP4	26	Female	Bachelor	Pharmacist	1
HP5	31	Female	Bachelor	Medical officer	4
HP6	32	Female	Bachelor	Medical officer	4
HP7	27	Female	Diploma	Occupational therapist	3

### Themes

The analysis yielded seven main themes and 22 subthemes explaining how early-career HPs in primary care clinics navigate and engage in IPC practices when managing diabetes (Table 2). The sub-themes may appear closely related but they represent distinct aspects of collaborative practice by participants.

**Table 2:** Summary of themes and subthemes.

Themes	Subthemes
(1) Initiating and continuing dialogue	Making the first move and bridging the gap
	Making referrals
	Discussing patient continuity of care
(2) Creating cohesiveness	Socializing and getting familiar with each other
	Respecting one another
	Building trust
	Learning one’s working styles and personalities
(3) Effective ways of communication	Sharing information through various ways
	Being tactful in communication
(4) Having own personal values	Valuing each other’s roles and capabilities
	Willingness to accept and improve on own mistakes
(5) Willing to work synergistically	Willingness to ask for help
	Moving away from hierarchical attitudes
	Co-sharing responsibilities and burdens
	Supporting each other
(6) Learning from each other	Openly informing about mistakes
	Organizing and attending interprofessional learning (IPL) activities
	Coaching peers to collaborate
(7) Embracing diversities and resolving conflict	Letting others speak and listen
	Employing current evidence in medicine-based practice
	Allowing disagreement without compromising patient safety
	Tolerating differences in other’s working skills and behaviors

### Theme 1: Initiating and continuing dialogue

Participants identified initiating dialogue as the foundational step and continuing the dialogues for ongoing multidisciplinary collaboration in managing diabetes, a complex health condition. This theme encompassed three subthemes: ***a) Making the first move and bridging the gap, b) Making referrals,*** *and* ***c) Discussing patient continuity of care.***

#### a) Making the first move and bridging the gap.

ECHPs recognized the need to take the initiative in establishing IPC rather than waiting passively. Participants emphasized the importance of actively reaching out to colleagues within the same facility to build rapport and foster collaborative relationships. By proactively initiating conversations and introducing themselves, ECHPs and their colleagues became familiar with one another, paving the groundwork for future collaboration and forming the foundation for ongoing professional relationships. This approach was considered essential for building confidence, enabling ECHPs as newcomers to negotiate care plans and address patient management gaps effectively.

*“I think initiating a high-impact conversation matters, because that’s where I see us [ECHPs] start to build self-confidence, ok…”* ***(HP7)***

*“When I first came here…went to visit all the occupational and physiotherapist units…gone in and have a look what kind of things that they have. For pharmacist, I do go in also to see who is actually in charge of diabetes patients, what kind of services they can provide, how frequent is their appointment with diabetes patients, so I think it’s on your own initiative to actually see what kind of service they can provide in your setting.”* ***(HP2)***

#### b) Making referrals.

Referrals emerged as another key context where dialogue played a critical role in explaining patient needs and ensuring appropriate care. Participants contacted multidisciplinary HPs through referral systems using written memos, standardized referral forms, or digital platforms such as WhatsApp, which is useful when face-to-face meetings were impractical due to high patient volumes. Sharing referral forms photos through WhatsApp helped expedite the referral process, ensuring prompt communication among relevant.

*“At least with WhatsApp group, when we refer a patient, we can take a picture of the form, update them in group chat.” (* ***HP6)***

Participants emphasized that referrals did not end at patient discharge. Ongoing dialogue remained necessary to reassess patient’s progress, initiate re-referrals when needed, and keep all HPs updated for coordinated and responsive care. As part of their responsibility for continuity of care, participants willingly reviewed patients when multidisciplinary colleagues initiate re-referrals.

*“Actually, if the doctor asked to re-counsel the patients or review something [re-referred same patient], we [multidisciplinary HPs] just do it. We do have our own schedule for patient counselling.”* ***(HP4)***

#### c) Discussing patient continuity of care.

Continuing dialogue after referrals through frequent care team discussions was essential. Participants reported that these ongoing team discussions helped to direct their own patients to the most appropriate professionals. These discussions often began informally with case consultations before formal referrals were initiated.

*“Oh basically, maybe they [other multidisciplinary HPs] will call me and enquire if this particular patient of theirs is suitable for me to be consult? If I say yes, then they will memo to the medical officer. On that memo, they will note that this patient should be referred to [required] occupational therapy services too.”* ***(HP7)***

Participants added that frequent team discussions allowed them to familiarize themselves with patient cases, reducing the need to repeatedly gather histories.

*“Through this kind of care [interprofessional collaborative care], since we [multidisciplinary team members] often do meetings [team discussions], we get to remember the patients more, and actually you can build a better relationship with the patient…” (* ***HP2)***

Some participants suggested holding brief discussions during breaks or end-of-workday sign-outs at clinic stations.

*“Because we [multidisciplinary team members in the clinic] have the same break time, leave the same time, and see patients at the same time... maybe before we punch out [sign-out], we can have a quick chat”* ***(HP6)***

Nevertheless, participants reported that time constraints remained a persistent challenge to maintaining consistent dialogue, despite their efforts to find opportunities for brief discussions.

*“Once, during a CME (Continuous Medical Education) presentation by the physiotherapist and occupational therapist, we actually tried asking if we can get, like an update on the patient [in the same care team]. But basically, they [the physiotherapist and occupational therapist] have a lot of patients to treat, so it’s sort of lack of time...”* ***(HP5)***

### Theme 2: Creating cohesiveness

According to the participants, building IPC requires gradually developing cohesive working relationships, starting with simple gestures like greetings before deeper engagement. This theme is further elaborated in four subthemes: ***a) Socializing and getting familiar with each other, b) Respecting one another, c) Building trust,*** *and* ***d) Learning one’s working styles and personalities.***

#### a) Socializing and getting familiar with each other.

Healthcare professionals (HPs) developed rapport through both informal and formal interactions. Participants suggested that informal social events such as family days or off-site gatherings helped break down barriers, creating a more relaxed communication environment. These interactions not only strengthened relationships but also provided opportunities to resolve misunderstandings by getting to know one another on a personal level. Over time, this facilitates smoother collaboration in clinical settings.

*“I think outside of the job scope we can do [organize] like Family Day, just to strengthen our bonds. That way, if any misunderstanding occur, maybe they [other multidisciplinary HPs] can understand us [ECHPs] a bit more [better]”* ***(HP1)***

Some participants appreciated formal events such as health programs for enabling multidisciplinary team discussions. Unlike daily patient management with limited team interaction, these event discussions offered them opportunities to interact with other disciplines HPs. Participants viewed these events as valuable for relationship-building.

*“…if we [multidisciplinary HPs] are holding [running] a program, during the discussion time, I have the chance to socialize or communicate with them [multidisciplinary members} from the other teams…if we don’t have a program, I am just [mainly] dealing with the Family Medicine Specialists (FMS) [Primary Care Physicians] and also the DE (Diabetic Educator) for diabetic management patients.”* ***(HP7)***

They further expressed that their relationships strengthened overtime, and they developed greater familiarity with one another’s skills and responsibilities. Hence, collaboration became smoother, leading to better patient care.

*“As time goes by [passes], if you’re [ECHPs] friendlier, they [when needing help from other multidisciplinary HPs] become OK… At first, you can sense their resistance [from other multidisciplinary HPs who later say things like] ‘Huh, doctor [HP2], why do this, why did you refer to me here and there...’”* ***(HP2)***

One participant expressed that senior HPs should take the lead in rapport-building with ECHPs. When senior staff welcomed newcomers, it generated a sense of belonging and team cohesion. This welcoming approach can allow junior team members to integrate smoothly and contribute effectively to the team.

*“First, seniors need to build rapport, don’t make juniors [ECHPs] feel scared, seniors [should] become their friends, so juniors feel close to them. Like when you [ECHPs] need to ask the pharmacist [senior multidisciplinary HPs] for help to get something for a patient, if you’re afraid of them, then teamwork can’t happen. But once you [ECHPs] know them [senior multidisciplinary HPs] and are close to them, you get used to them, and it just feels normal, right? No ‘you you or me me’ divisions.”* ***(HP3)***

However, participants cautioned ECHPs need to avoid personal conflicts, stressing that maintaining good working relationships was essential for harmonious collaboration.

*“Try to build a good relationship with everyone [HPs in primary care clinics] and try not to involve in personal conflicts, so that it will help you [ECHPs] to work peacefully.”* ***(HP5)***

#### b) Respecting one another.

Team cohesiveness develops when everyone respects each other, treating colleagues with dignity, appreciating each other’s expertise, and creating space for every opinion to be heard. Some participants shared that their respect was accompanied by trust in multidisciplinary HPs’ specialties, decisions and opinions in patient care management. They accepted one another’s professional capabilities and trusted that team members knew what they were doing for the patients they were caring for in the same team management:

*“Uh, I don’t disagree because I do respect their [multidisciplinary HPs] specialties. Of course they know the patient better unless I notice something alarming, then I’ll let them know. But I don’t really disagree [trusting multidisciplinary HPs’ capabilities] because they know their specialties better than me.”* ***(HP2)***

Additionally, respecting one another to work collaboratively is accompanied by letting others speak and actively listening to their opinions rather than completely shutting them out. The participants further shared that when acquiring help and knowledge in other fields of practice where they were lacking, they allowed other HPs to speak while they became active listeners. The participants further mentioned that they respected the other fields’ HPs’ explanations which were accompanied by protocols that were trustworthy.

*“If I [a medical officer] think the first choice [e.g., a medication] is better, I’ll put it forward [informed decision to, e.g., pharmacist]. I’ll say [to pharmacist]: ‘This is the first choice, and this has to be the choice.’ So, usually there’s no problem with that. But if there are certain things I don’t know, if I’m not too sure and they [other multidisciplinary HPs, e.g., pharmacists] have more knowledge on drugs, then there’s nothing wrong with listening to them [other multidisciplinary HPs, e.g., pharmacists] because they usually will explain [e.g., medications] with protocols in their hands.”* ***(HP5)***

#### c) Building trust.

Early-career HPs frequently encounter skepticism about their clinical competencies. Participants reported needing to build trust over time by repeatedly proving their skills and commitment. As they established rapport with colleagues, gaining trust became easier.

*“…they [other HPs] have no trust when you are fresh graduated [ECHPs]…I proved myself, I showed myself, not because of I want praises or anything…But I am doing the best because of wanting to deliver the best of ideas [cares]…So with time, they [other HPs] looked at me like ‘oh OK, trust, I can be trusted’”* ***(HP7)***

*“Here, I know them [other HPs] better, so they find it easier to trust us [ECHPs], and we find it easier to trust them too”* ***(HP4)***

#### d) Learning one’s working styles and personalities.

Participants identified the importance of understanding other HPs’ task approaches and communication styles to maintain cohesiveness. By learning each other’s working styles and personalities, they could adjust their interactions to enable more effective teamwork. Participants mentioned they learned their colleagues’ personalities and work practices through, for example, having casual conversations during lunch breaks, a process that facilitated smoother collaboration.

*“Oh like mix with them [other HPs], chat with them more often, have lunch together, have simple talk, things like that. They get to know you [ECHPs] better, they see you are not a fussy person, and they are ok with you.”* ***(HP2)***

Some preferred observing colleagues in meetings or events to learn how they communicated and reacted to different situations. This observation helped them approach colleagues more confidently, knowing how best to engage with them.

*“Because I feel I’m trying to look for [figure out] the person’s [other HPs’] personality pattern. If I know what kind of person they are, I’ll have more confidence to say…to speak out what am I thinking, like that.”* ***(HP7)***

### Theme 3: Effective ways of communication

With rapport established, communication becomes central to teamwork in IPC. Participants emphasized the importance of both verbal and written communication and adapting communication styles to different situations. This theme is summarized in the following two subthemes: ***a) Sharing information through various ways,*** and ***b) Being tactful in communication****.*

#### a) Sharing information through various ways.

Participants explained that WhatsApp was widely used for real-time updates, enabling HPs to keep each other updated on patient progress efficiently. Many viewed WhatsApp groupchats as an efficient tool to avoid miscommunication, as information could be easily referenced when consulting patients.

*“I think every profession should have this [Whatsapp group with multidisciplinary HPs]. The easiest way now is a WhatsApp group. At least with a WhatsApp group, when we [HPs] refer a patient [to other discipline HPs] and check [leaving a request in Whatsapp group] ‘Can you later inform me about the patient’s progress?’ … Then when patient’s progress report comes back to us, we can use that as references [probing other discipline HPs], ‘Patient still complaining of this, can you see what else can be done?’, something like that.”* ***(HP6)***

However, participants also highlighted the potential for misunderstandings due to tone, as well as concerns about patient privacy and data security, though these were considered unavoidable challenges in ensuring the delivery of optimal patient care.

*“…sometimes whatever you’re saying in WhatsApp, the tone might not reflect the actual one. That can be miscommunication.”* ***(HP5)***

*“Yeah, patient information is actually seen by everyone in the group at that time. But, if shared with limited info, that will cause inadequate treatment as well. So, I think this is for treatment purposes. I don’t think there should be any problem with me sharing my patient’s info with the team because they [multidisciplinary HPs in the care team] will receive the form [referral] and the information will go to them as well. So, the issue here is just from paper to the web [WhatsApp group], that’s all.”* ***(HP6)***

From another perspective, participants shared how WhatsApp groups served as platforms to raise ECHPs’ visibility, initiated by seniors from primary care. The seniors, such as primary care physicians, promoted individual ECHPs based on familiarity, respect and trust for their skills and performance. ECHPs earned trust and respect over time by consistently demonstrating competence, eventually gaining more referrals and collaborative opportunities.

*“I try to develop work processes in the health clinic. When the FMS [Primary Care Physician] from other visiting clinic, where I’d worked in, mentioned my name [HP7] in the bigger group [district-level WhatsApp group], so at that moment, the others [the multidisciplinary team members in the district-level WhatsApp group] seemed to think, ‘Okay, she [HP7] can do this [capable of handling specific patient’s needs].’ I noticed that afterward, other medical officers started asking me [other multidisciplinary HPs seeking HP7’s services for their patients]…’Are you able to do this [consult on specific patient’s needs]?’”* ***(HP7)***

Meanwhile, online meetings provided flexibility for those unable to attend in person, though participants acknowledged that in-depth patient discussions often required direct communication.

*“…we [multidisciplinary HPs] can just come meet up…like in an online meeting, or in a seminar room, not all of us, just some...to discuss for better management for diabetic patients.”* ***(HP1)***

*“We actually tried virtually…during a presentation Q&A (questions and answers) session…So we asked there, but I think we might need more direct discussion.”* ***(HP5)***

Participants indicated that phone calls were a quicker, more direct method for discussing complex patient cases, particularly when dealing with sensitive information. While WhatsApp was convenient for general updates, phone calls allowed for more complex and confidential discussions.

*“But if there’s something more to discuss [with the multidisciplinary HPs in the team], maybe relating to patients, family support, or the patients’ mental condition, I think should call directly [for discussions].”* ***(HP5)***

Some participants shared that face-to-face communication remained preferable for resolving any potential misunderstandings.

*“Better communication and better understanding [help] avoid any misunderstanding or arguments. We [HPs] can argue face-to-face or just have some face-to-face chit-chat…lesser hard feelings if face-to-face [meet in person], I think.”* ***(HP1)***

Face-to-face meetings are effective for urgent patient issues requiring immediate attention. Participants expressed that frequent face-to-face meetings enabled real-time case discussions with open dialogue. Unlike written or digital communication, these interactions allowed for instant clarification of doubts and observation of nonverbal cues which further foster friendship and openness within the team, enhancing collaborative effectiveness.

*“I do prefer if the thing is urgent…can meet me face-to-face [for discussion].”* ***(HP7)***

*“I think there’s no problem with communication in closed meeting. When you’re close, you feel happy [at ease] with that person…and you don’t have any problems with them [other HPs].”* ***(HP3)***

Participants shared written communication was also critical. Concise, jargon-free notes ensured accessibility across disciplines, enabling multidisciplinary HPs to quickly grasp relevant information without parsing lengthy documentation for positive collaborative teamwork.

*“… they [different multidisciplinary HPs in the same team] can come back [respond] with something more compact [after seeing a patient under the same team care], like a feedback form for us [HPs in the team care]. It’s not writing essays [style]. This way, we know where to look [check] quickly rather than reading through everything.”* ***(HP6)***

*“For me personally, I write it in full terms. I’m don’t use shorthand or jargon …so I hope they [multidisciplinary HPs] will understand what I’m trying to convey.”* ***(HP7)***

#### b) Being tactful in communication.

Participants stressed the importance of tailoring language when requesting assistance or addressing sensitive matters. They adjusted wording based on team members’ personalities to ensure receptiveness. Otherwise, they sought help from other colleagues where they brainstormed to determine the best way to convey messages.

*“if the person [individual team members] is approachable… I will just speak my opinion. But then if the person kept questioning me, I still speak up, though it will take longer time to think [decide] what to say, so they [other team members] will agree. And if they don’t agree…I will brainstorm with others like my colleagues, to find the best or proper words to express myself.”* ***(HP7)***

One participant highlighted that communicating with more senior colleagues who had made errors in medical records or patient management was challenging. This required a delicate approach, politeness, and professionalism to resolve issues without causing friction. Apologizing for any disruptions was suggested to smooth interactions and maintain positive working relationships.

*“...even if they [senior HPs] made a mistake, it’s okay, we apologize first because we’re the ones disturbing [interrupting] them, right? Sometimes they’re talking to a patient, and we [ECHPs] interrupt them. So, why not apologize? That way, they are opened to acknowledge [admit] their mistake as well, and we can manage the case together.”* ***(HP4)***

### Theme 4: Having own personal values

Practicing positive personal values emerged as fundamental to effective IPC. Participants described how values such as respect, support, and trust influenced individual behaviors, professional relationships, and workplace dynamics. The absence of these values could undermine IPC efforts, affecting teamwork and patient outcomes. Within this theme, participants highlighted several key subthemes: ***a) Valuing each other’s roles and capabilities,*** and ***b) Willingness to accept and improve on own mistakes***.

#### a) Valuing each other’s roles and capabilities.

Participants emphasized the importance of recognizing and valuing HPs’ unique contributions and skills within the team. Acknowledging these roles boosted motivation, job satisfaction and workplace positivity. When ECHPs received praise for their work, it validated their contributions and instilled a sense of belonging.

*“…friends [other HPs in primary clinics] will also tell me ‘Oh, you saw this patient a few months back, you added these medications. Now, the liver function tests are getting better, and their sugar [levels] is getting better.’”* ***(HP2)***

When seniors valued junior colleagues’ effort, their positive feedback served to validate the juniors’ professional competencies while simultaneously encouraging other team members to trust junior’s capabilities. This recognition naturally generated more referrals and collaborative opportunities to enhance patient care through effective utilization of ECHPs’ unique strengths. Concurrently, this dynamic provided ECHPs’ superiors with valuable opportunities to observe and track their junior staff’s development.

*“I try to develop work processes in the health clinic. When the FMS [Primary Care Physician] from other visiting clinic, where I’d worked in, mentioned my name [HP7] in the bigger group [district-level WhatsApp group], so at that moment, the others [the multidisciplinary team members in the district-level WhatsApp group] seemed to think, ‘Okay, she [HP7] can do this [capable of handling specific patient’s needs].’ I noticed that afterward, other medical officers started asking me [other multidisciplinary HPs seeking HP7’s services for their patients]…’Are you able to do this [consult on specific patient’s needs]?’ My supervisor told me about this situation.”* ***(HP7)***

Additionally, valuing others extended to recognizing how different professions complement each other’s skills. For instance, pharmacists frequently uncovered medication adherence issues that patients hesitated to share with doctors. Consequently, medical officers depended on pharmacists’ nuanced understanding to uncover why a patient had discontinued treatment. This interprofessional appreciation helped to connect knowledge gaps and reinforced the significance of each role within the team.

*“OK, so we [medical officers] refer them [patients] to pharmacist with reasons of referral as such: ‘why did patient refused to follow this [medication plan] [The patient] works as a lorry driver, [eats] late breakfast…did not want to take the medication?’ Then an example the pharmacist will respond to us [medical officers] in an attached evaluation form in patient card [medical record]: ‘Before the breakfast, he [the referred patient reviewed by pharmacist] was afraid of hypoglycemia… takes the medication wrong way.’ So, during the next review [patients revied by medical officers], medical officers should see the form as well [read through the written responses/reports by other HPs].”* ***(HP6)***

#### b) Willingness to accept and improve on own mistakes.

Acknowledging one’s own mistakes and reaching out for help were perceived as ways to strengthen professional relationships and open avenues for growth. These growths were achieved through self-seeking guidance from senior colleagues, conducting personal research, and consulting existing evidence-based guidelines such as the Clinical Practice Guidelines (CPGs). This approach not only improved their competencies but also reassured team members of their dedication.

*“There were some disagreements [with other team members] … We [ECHPs] referred back to the resources [guidelines or seniors] on the same management plan until it was accepted [by the disagreed team members]…if I was wrong [on my management knowledge], I studied and so did they [team members studied too]”* ***(HP6)***

Participants shared that embracing openness to constructive feedback and a willingness to adapt their practices based on others’ input built and maintained harmonious collaborative relationships and a productive work environment.

*“I got no problem for them [other HPs in the team] correcting me… sometimes when I made mistakes with the prescriptions, the pharmacist will just come and look for me. I wouldn’t give them any hard time or anything.”* ***(HP2)***

### Theme 5: Willing to work synergistically

Participants emphasized the need for an inclusive and cooperative approach to patient care, where every team member, regardless of seniority or discipline, is willing to work synergistically. This theme is supported by four subthemes: ***a) Willingness to ask for help, b) Moving away from hierarchical attitudes, c) Co-sharing responsibilities and burdens,*** *and* ***d) Supporting each other.***

#### a) Willingness to ask for help.

Humility and openness to seeking guidance were necessary traits in navigating the complexities of healthcare settings. Participants emphasized that ECHPs should approach senior colleagues with clear, professional communication and without hesitation. Additionally, when awaiting responses, they should remain patient and respectful, understanding one another’s time constraints.

*“When I’m unsure how to refer patients for simple things like a mammogram or pap smear, I ask, ‘OK sister [a nurse], how do I refer for this? Which form should I use…where does the take this’ Most seniors [other HPs] here have worked longer than I have, so they know the pathway better [procedures in the clinic]…so I ask anyone else [different discipline HPs] too”* ***(HP6)***

#### b) Moving away from hierarchical attitudes.

The IPC environment should not be constrained by traditional hierarchies. Doctors, often perceived as gatekeepers of care, were encouraged to actively listen to and respect multidisciplinary HPs’ input. Conversely, non-medical doctor ECHPs were advised against automatically deferring to doctors’ opinions, instead confidently asserting their expertise.

*“I feel that when we [non-medical doctor ECHPs] work, we don’t follow professions [professional hierarchy] like, ‘oh, you’re a doctor, you’re superior; we must agree, we must say yes [to a doctor].’ I don’t think that’s right, because we [all professions] work together. When we work, we viewed everyone equally. So, if you’re [non-medical doctor ECHPs] right, then you’re right, and, mistakes should be accepted, that’s how it is. I don’t think we should look up to someone just they’re a doctor. Similarly, we shouldn’t look down on nurses, thinking ‘oh, they’re wrong.’ I’m not saying pharmacists are superior either. Everyone has their own expertise.”* ***(HP4)***

Participants acknowledged that dismantling hierarchical attitudes began with building positive relationships and learning from each other’s strengths and weaknesses, which helped create a culture where everyone felt valued.

*“If you just stay in your position and do not want to socialize with others [other HPs], it will be difficult for you to work with them [other HPs in the team] in future because by nook or crook, you’ll have to implement patient management together with the team.”* ***(HP7)***

#### c) Co-sharing responsibilities and burdens.

Co-sharing responsibilities involved collaborating to address patient needs holistically. Participants described how different disciplines managed various aspects of patient care, collectively filling gaps and ensuring comprehensive management. For example, one participant explained that when a patient required intervention beyond their expertise, they would engage the relevant specialists, such as physiotherapists, and coordinate care effectively. Understanding and supporting each other’s roles was essential for this synergy.

*“…we [medical officers] try our best to do [assessed patient and delivered medical care] what is necessary…When physiotherapy is indicated [during medical assessment], we refer them [patients] to the physiotherapist. But usually, we also try to convince them [patients]to go to them [physiotherapists] and explain how they [patients] can actually see improvements.”* ***(HP5)***

This co-sharing of responsibilities extended to strategizing patient care for future team members and recognizing obstacles other HPs might face in meeting patients’ needs. Participants exemplified this by delaying referrals to other HPs for overwhelmed patients, ensuring care delivery was paced according to each patient’s capacity to process information.

*“So usually, for the first visit, I would not refer [to multidisciplinary HPs]…because during the first visit where we [medical officers] explain all about the disease and start medication. Sometimes patients can’t absorb too much [information] at once. So, what I usually do is I schedule appointment [a follow-up] within a month to see their progress, then I’ll refer to the dietitian on the second visit. The 1st visit, patients can be overwhelmed with information.”* ***(HP2)***

Co-sharing burdens included actively helping and managing team members’ workloads to promote team well-being. Participants shared that they covered for others during high-stress periods or stepped in to help with complex cases beyond their formal duties. They co-shared such burdens without expecting reciprocation, motivated instead by a genuine commitment to maintaining a cohesive team environment.

*“Actually, we [HP7 and diabetes educator] do work together…when we have the health programs like patient education. But when there’s no program, I still work together with her [diabetes educator], just not simultaneously. I might help her with foot screenings and then share her copies of the patients’ results.”* ***(HP7)***

Participants particularly expressed senior HPs making personal sacrifices, such as forfeiting their own breaks to assist ECHPs, demonstrating how compassion and friendship strengthened professional relationships.

*“Even if I need any information outside working hours, they [senior HPs] still do provide me with that or during the working hours or break time. During the first five months…I don’t get lunch breaks… I see patients until 6:00 PM.”* ***(HP5)***

#### d) Supporting each other.

Participants described supporting each other, particularly in healthcare’s emotionally demanding environment. Providing emotional support to colleagues, whether by listening or helping during difficult periods, helped sustain team morale. They valued senior colleagues’ support in navigating new workplace pressures. Knowing others were available to assist with both personal and professional challenges reinforced a sense of unity and shared purpose.

*“Yes they [senior HPs in the clinic] did, actually, the most important thing that they gave me was emotional support. So after that, I became very comfortable with them.”* ***(HP5)***

They also reflected on the importance of understanding one another’s workloads and limitations, suggesting that support should be offered with sensitivity to individual capacities and schedules. Participants viewed selfless and open-hearted assistance as essential for successful collaboration, creating a culture where all team members felt truly united rather than isolated.

*“I feel that I can give them [other multidisciplinary HPs] something to work with, but I don’t know whether they can actually cope with it or actually do it. Yeah, because I understand they also have limitation.”* ***(HP6)***

*“I’m happy when no one acts selfishly, when everyone works as a team. Even though they are from different professions, it feels like one family.”* ***(HP3)***

### Theme 6: Learning from each other

Learning from each other fosters effective IPC since healthcare professionals rely on each other’s expertise to enhance patient care, and this mutual education helps bridge gaps in knowledge and practice. This theme comprises three subthemes: ***a) Openly informing about mistakes, b) Organizing and attending interprofessional learning (IPL) activities,*** *and* ***c) Coaching peers to collaborate.***

#### a) Openly informing about mistakes.

Participants highlighted the importance of addressing mistakes openly and constructively to maintain patient safety and improve care. When errors were identified, they should be communicated directly to affected individuals in a friendly, non-judgmental manner.

*“Yeah, if I feel something is right, we [ECHPs] must stand by it. Just inform the doctor. There’s nothing to hide because we’re prioritizing the safety of the patients, that’s our first mission. If the doctor makes a mistake, we have to inform them.”* ***(HP4)***

#### b) Organizing and attending IPL activities.

Engaging in IPL activities, such as continuous medical education (CME) sessions and brief multidisciplinary attachments, was valued as an effective way to educate and learn from one another. Participants remembered attending sessions to better understand other specialties’ services, including referral processes and best practice guidelines.

*“The physiotherapist, they gave a CME to us, the medical officers, regarding the services provided here in the primer [primary] clinic…we have some attachment with physiotherapy clinic…the physiotherapist’s session…we get the whole morning to watch them and learn – they provide this kind of therapy for this kind of patients…”* ***(HP1)***

*“Ok, so if the doctor keeps repeating the same mistakes, then we [pharmacists] can organize a CME on prescribing practices... we can bring it up to them [the doctors] again.”* ***(HP4)***

#### c) Coaching peers to collaborate.

Coaching peers to collaborate was identified as an effective method for fostering collaboration, particularly among ECHPs or those new to a clinical setting. Senior staff or more experienced colleagues guided their junior peers in communication and collaborative practices. Participants shared that coaches demonstrated how to interact with other professionals, introduced newcomers to the team, and provided opportunities to observe interprofessional interactions.

*“Can invite us [ECHPs] along so we can observe how things are done, how to communicate with other care workers since our seniors already been working there…we learn from watching how our colleagues do things too…We observe our bosses – how they handle cases, their attitude, and we actually learn from that as well”* ***(HP4)***

### Theme 7: Embracing diversities and resolving conflicts

Participants acknowledged that diversities and conflicts among team members are inherent aspects of teamwork in IPC. Strategies for managing diversities and conflicts are summarized in the following subthemes: ***a) Letting others speak and listen, b) Employing current evidence in medicine-based practice, c) Allowing disagreement without compromising patient safety,*** *and* ***d) Tolerating differences in others’ working skills and behaviors***.

#### a) Letting others speak and listen.

Active listening and providing space for others to voice their opinions were seen as fundamental in resolving conflicts. Participants stressed the importance of considering colleagues’ perspectives before presenting their views during decision-making. By allowing others to speak and practicing open-mindedness, team members could facilitate complete information exchange and address issues collaboratively.

*“…when you work as a team, everyone should have a right to voice their opinion...For example, things that the doctor may miss, but the nurse is able to catch...[On the other hand] The pharmacist has caught some problems too, then we [multidisciplinary HPs in the team] may be able to link that – actually this arises from the same issue.”* ***(HP2)***

#### b) Employing current evidence in medicine-based practice.

Referencing established clinical guidelines helped support the ECHPs’ care proposals and facilitate consensus among team members. ECHPs reported that using guidelines had helped validate their suggestions and subsequently fostered trust with senior team members. This practice strengthened their arguments and promoted adherence to best practices within the team.

*“So what I do is, I open the CPG (Clinical Practice Guidelines) and show, ‘Doctor, here’s the evidence, this is why these medications can’t be used together,’ and the doctor believes it. As a fresh graduate [ECHPs], just starting work, sometimes people find it hard to trust us [ECHPs]. So, when we encounter situations like this, we show the evidence of what we’ve studied, present the proof.”* ***(HP4)***

Others sought advice from senior or more experienced colleagues to manage conflicts. Participants shared that involving a third party could help mediate disagreements and provide additional insights. Seniors often acted as moderators, assisting in interpreting guidelines and offering solutions based on their expertise. This collaborative approach fostered learning and supported professional growth among ECHPs.

*“And then I [a pharmacist had contacted a junior medical officer about the unsuitability of a medication prescription] just tell the pharmacist ‘It’s OK, just let me consult my superior [the FMS – Primary Care Physician]…’ and at that time my superior said, ‘Oh, actually no problem with the medications [being prescribed by junior medical officer].’ So to me, I’m OK if there’s a conflict [in practice]. We [junior medical officers] just refer to the guideline – his [pharmacist] guideline and my guideline contradict, we [junior medical officers] just seek opinion from my FMS [Primary Care Physician].”* ***(HP2)***

#### c) Allowing disagreement without compromising patient safety.

Participants recognized that not all conflicts could be resolved immediately or meet everyone’s satisfaction, given the diversity in clinical practices. Paradoxically, they agreed to proceed with decisions that did not compromise patient safety, even when those decisions conflicted with their preferred approaches. These disagreements were revisited to discuss differing viewpoints and align understanding.

*“If they [doctors] say, ‘mmm, I don’t want to,’ or maybe they disagree, then it’s okay...we [ECHPs] can discuss it next time…Because sometimes, if the doctor insists, we just go along with it, as long as it doesn’t burden the patient and ensures the patient’s safety…” (* ***HP4)***

#### d) Tolerating differences in other’s working skills and behaviors.

Participants recognized that colleagues might have varying levels of skills and experiences, communication styles, or personal habits that could impact teamwork. By tolerating these differences and focusing on professional interactions, team members were able to maintain harmonious relationships while resolving and preventing conflicts.

*“if the doctor doesn’t like this officer [other HPs] interrupting them…maybe they [the doctors] feel annoyed because they [other HPs] don’t know how to communicate with each other, but actually people say that since we’re [all HPs] working together under the same roof [in the same primary clinic], we actually could just accept it [other HPs’ skills]”* ***(HP4)***

Participants with better work experiences shared that seniors provided them with mentorship and support during skill struggles, fostering mutual growth and understanding. They also emphasized that maintaining professional work relationships and addressing issues transparently were key to achieving common goals.

*“OK, so I think they [seniors] see me as a freshie [ECHP] who needs to be monitored and guided. So I don’t think that they misuse me or mistreat me.”* ***(HP6)***

*“Yes, they [other multidisciplinary team members] received my ideas and can accept them [the ideas]… and tolerate [HP3’s differences]. There’s a sense of give and take… When there’s a discussion, we [the multidisciplinary HPs] work as a team, we agree, and if there are any suggestions from the doctors, we hold a meeting to address them [the suggestions from the doctors].”* ***(HP3)***

## Discussion

This study explored early-career health professionals (ECHPs) experiences navigating interprofessional collaborative care (IPC) in diabetes management within primary care clinics. Seven main themes with detailed subthemes emerged and are deeply interrelated, demonstrating that IPC is complex to practice, particularly for early-career individuals entering independent clinical practice.

Initiating and continuing dialogue served as the foundational catalyst in collaborative practices. Participants emphasized that “making the first move” was the key to overcoming professional barriers and building team relationships. This proactive engagement facilitates collaborative skills and creates cohesiveness [10,69]. A dialogue context through referral-making required dismantling professional silos to ensure timely processes that safeguard patient safety and continuity of care [5,70,71]. This finding contrasts with previous studies where new health professionals (HPs) hesitated to engage experienced colleagues due to perceived power imbalances [72–74]. While Malaysia’s cultural norms of respecting authority figures may amplify such hesitations [75], our participants recognized the need to overcome this barrier to address patients’ needs [76]. This finding suggests a shift toward egalitarian collaboration.

Referrals were not mere transactional handoffs but required ongoing dialogue to ensure continuity. Participants stressed the importance of post-discharge discussions and re-referrals to prevent care fragmentation, aligning with D’Amour et al.’s (2008) concept of “relational continuity” [77].

The current referral processes were facilitated by digital platforms (e.g., WhatsApp). This finding reflected broader healthcare trends, where instant messaging platforms were heavily relied on, especially in IPC care. These platforms enabled real-time information exchange [78–81], but raised concerns about breaches of patient confidentiality and data security [78,82].

Individual initiatives to initiate and continue dialogue contributed to creating cohesiveness within the team. These working relationships were strengthened through formal or informal social interactions, as well as through becoming familiar with each other, which helped overcome professional barriers and facilitate unity among team members. Our participants emphasized that senior health professionals could further reinforce these working relationships by taking the lead in building rapport with ECHPs. In the literature, such social cohesion roles is shown to enhance interprofessional relationships and collaborative practices in day-to-day clinical practice, particularly within Malaysia’s collectivist professional culture [75,83–88].

Respecting one another and building trust are required to create team cohesiveness, ultimately improving job satisfaction, and reducing workforce turnover [89–91]. Similar to previous studies’ findings, ECHPs needed to build respect and trust through a gradual process of demonstrating consistent competence and commitment [88,92,93]. When they felt connected and respected, they were more likely to communicate openly and constructively. Learning about colleagues’ working styles and personalities enabled members to adapt and personalize their interactions [2,94–97]. This personalization helped anticipate their responses and reduced misunderstandings during collaborative care, especially when embracing diversity and resolving conflict [94,96,98]. Our findings addressed a literature gap regarding ECHPs interpersonal dynamics in IPC, supporting the notion that strengthening relationships through relational coordination is essential for advancing healthcare quality [1,2,98].

These cohesive relationships enhance communication effectiveness in IPC. Participants leveraged multiple communication channels including digital, face-to-face and written notes, to facilitate information sharing and decision-making. Digital platforms, such as WhatsApp groups, enabled rapid updates and acted as visibility platforms for ECHPs, aligning with social proof [99,100]. On the other hand, participants preferred face-to-face discussions for complex cases, emphasizing the importance of nonverbal cues and immediate feedback [101,102]. However, time constraints limited face-to-face interactions, posing a challenge in IPC. Hence, the heavy reliance on digital platforms for interprofessional collaboration requires further research to ensure and maintain patients’ confidentiality [78,79,81,82]. As for written notes, they should be concise due to time constraints and with clarity across disciplines.

Participants expressed that effective communication requires clarity and sensitivity to emotional and cultural contexts, along with mutual respect and understanding of different professional cultures [76,103]. When encountering sensitive situations, such as addressing mistakes made by senior colleagues or communicating with challenging personalities, participants recommended employing tactful language (e.g., an apology and carefully crafted words) and/or seeking colleagues’ assistance. These strategies help maintain professional relationships and prevent misunderstandings. Therefore, communication training should include cultural competence and emotional intelligence [15,104–107].

This study revealed that having personal values fostered a cohesive environment, learning from each other, embracing diversity, and resolving conflicts. These personal values involved valuing each other’s roles and capabilities as well as a willingness to accept and improve on own mistakes. Participants expressed that acknowledging and appreciating each professional’s unique expertise in patient care not only promoted a positive work environment, but also enhanced job satisfaction, bridged gaps in patient care and ultimately led to gaining mutual respect among team members [19,92,108–111]. The participants’ willingness to accept and improve on own mistakes and uphold personal values reflected a growth mindset and psychological safety. They perceived challenges and failures as opportunities for self-development while feeling secure enough to express themselves without fear of negative consequences [17,97,112–115]. This mindset is essential for continuous improvement and effective team functioning in IPC.

Our participants demonstrated a willingness to work synergistically to practice collaborative care, moving beyond traditional hierarchical attitudes through these interconnected components of effective communication, cohesive relationships, practicing personal values and learning from each other. They actively contributed to decision-making processes with a sense of shared responsibility by seeking help from seniors with openness and humility. This approach helped remove the traditional healthcare hierarchy that perceives medical doctors’ authority as superior to that of other healthcare disciplines [75,110–112,116]. They forewent a competitive approach while acknowledging and leveraging one another’s strengths to provide comprehensive and coordinated services, which could prevent additional relational conflicts in the future [24,111,117].

However, changing hierarchical attitudes remains challenging due to Malaysian cultural norms regarding authority and respect [75,83,84]. Sasagawa and Amieux (2019) found that collaborative efforts required professional competence and dispositional humility to foster an environment characterized by fairness, equality, transparency, non-punitive responses to error reporting and a supportive atmosphere [118]. In the emotionally demanding environment of primary healthcare clinics, collegial support was important, especially for ECHPs, who often relied on more experienced colleagues for guidance and emotional support [108,119,120]. This support system helped alleviate work pressure and fostered a sense of belonging within the team, ultimately maintaining the team’s morale. Therefore, organizing training programs that encourage participative management and shared leadership for both junior and senior HPs in primary health clinics, supported by organizational policies promoting IPC, could facilitate and sustain this cultural shift [103,121].

Learning from each other serves as both an outcome and a catalyst for cohesive relationships, effective communication, willingness to work synergistically, and the ability to embrace diversity and resolve conflicts. Openly informing about mistakes in a constructive, respectful, and non-judgmental manner can help build team competency, fostering a culture of safety, continuous learning, and improvement [114,122]. This approach also reflects a just culture, where errors are seen as opportunities rather than reasons for blame, thereby reinforcing transparency and accountability in healthcare [123–125]. Organizing and attending interprofessional learning (IPL) activities provided valuable opportunities to bridge ECHPs’ practice gaps, as they promote knowledge exchange and a shared understanding of roles and responsibilities that may prevent conflicts [10,14,103]. Additionally, participants highlighted that seniors should coach peers to collaborate, especially ECHPs. Such mentorship and role-modelling efforts help to: (i) improve ECHPs’ ability to engage and negotiate in interprofessional interactions and (ii) build familiarity and trust within the team [87,103,119,120].

Finally, embracing diversity and resolving conflicts are interwoven within all the components, enabling ECHPs to navigate disagreements since teamwork inherently involves varying working styles and perspectives. Participants shared that to resolve disputes, one must allow others to speak and listen to promote open dialogue and mutual understanding. Beunza (2013) mentioned that empathic relations emerge when team members can express and find value in one another’s ideas or actions [126]. Alternatively, our participants utilized established clinical guidelines or sought input from more experienced colleagues as conflict mediators to facilitate consensus and reinforce adherence to best practices [55,127,128]. When conflicts remained unresolved, our study found that participants allowed disagreement in their treatment plan without compromising patient safety, as they prioritized patient outcomes over their preferences. ECHPs sometimes needed to adjust their expectations while accepting diverse approaches to patient care, a process demanding professional maturity. Participants further highlighted the critical role of tolerance and flexibility in accommodating different working styles and behaviors. Embracing these differences reflects emotional intelligence and adaptability, which are essential in collaborative working environments [89,114,129].

This study discovered that participants are willing to engage in difficult conversations to resolve conflict, in contrast with previous findings where early-career health professionals avoided conflict due to fear of repercussions [127]. As many literature sources focus on hospital settings, our study has contributed valuable insights into and addressed the gap in knowledge of how primary healthcare early-career health professionals manage conflicts in IPC [89]. Conflict resolution training in professional development programs should cover negotiation, mediation, and assertive communication skills to empower ECHPs to accept and manage conflict effectively [89,110,114,126].

### Strengths and limitations

A key strength of this study is its in-depth qualitative exploration of early-career healthcare professionals’ experiences in primary care settings. Through rigorous thematic analysis incorporating constant comparison, seven interlinked important themes were identified (see Table 2) that offer a holistic perspective on the multi-faceted nature of IPC [28,33,130]. The constant comparative method enhanced the analytical depth and systematic development of themes, ensuring robust code refinement and theme development while identifying subtle patterns and relationships in the data [47,131]. While our findings showed detailed subthemes might appear granular, this approach was essential for capturing the complexity of collaborative practices over parsimony [29,30,49,52,53,132]. This granular approach aligned with contemporary recommendations where analytical richness is preserved rather than forcing premature theoretical closure, as consolidating related subthemes poses a risk of losing important practical distinctions that represent different aspects of collaborative practice [29,30,49,132].

Another significant strength lies in our study population of early-career health professionals working in primary healthcare clinics. While much of the existing literature focuses on hospital settings or final-year health students receiving IPC intervention during their internships, this study offers novelty by addressing a critical knowledge gap in investigating junior phase of collaborative care after leaving internships [15–17,22,133–135]. Furthermore, the inclusion of diverse healthcare professionals (e.g., medical officers, pharmacists, and allied health professionals) provides a multidisciplinary perspective on IPC, strengthening the trustworthiness of our findings.

While our study provides valuable insights, there are several limitations. The findings are specific to the Malaysian healthcare context, which has distinct cultural and organizational characteristics that may influence interprofessional collaborative practices. The inclusion of only two primary care clinics as study sites may limit the generalizability of our findings. Additionally, participants’ responses may have been influenced by social desirability bias, where they responded they believed were more socially acceptable. Although reflexive and iterative analytic techniques were implemented, the complete elimination of such biases is challenging in any qualitative inquiry [130]. Therefore, caution should be exercised when applying these results to different geographical regions or healthcare settings [136].

## Conclusion

This study explored how early-career health professionals (ECHPs) in Malaysian primary care settings navigate interprofessional collaborative care (IPC) in diabetes management, revealing that successful IPC relies on interrelated components. Key findings revealed that initiating and continuing dialogue was fundamental for overcoming professional barriers, with ECHPs actively engaging with team members despite traditional hierarchical norms, reflecting a shift toward egalitarian teamwork. While digital platforms facilitated collaborative coordination, concerns persisted regarding patient confidentiality, and face-to-face interactions remained preferrable for complex cases. Team cohesion emerged through shared leadership, conflict resolution strategies, and interprofessional learning (IPL), enabling ECHPs to adapt to diverse working styles and contribute confidently to decision-making.

The findings suggested both education and practice. Educational institutions should design IPC curricula that emphasize emotional intelligence, negotiation skills, and digital communication ethics to bridge theory-practice gaps. Healthcare institutions, meanwhile, must reinforce collaborative practices through organizational policies and senior support, including integrating collaborative components into structured mentorship programs, IPC training initiatives, and fostering a culture of effective communication and shared values.

Critical gaps for future research include examining the role of informal socialization, peer coaching and the long-term impact of digital communication on IPC. These gaps should be addressed through targeted research and policy reforms to better support ECHPs in navigating collaborative diabetes care in primary care settings.

## Supporting information

S1 File Respondent information sheet. (DOCX)

S2 File Informed consent form. (DOCX)

S3 File Interview topic guide. (DOCX)
