# Supplementary material for: Navigating interprofessional collaboration in diabetes care: A qualitative study of early-career health professionals in malaysian primary care clinics
Source: PLoS One. 2025 Oct 28;20(10):e0335192. doi: 10.1371/journal.pone.0335192 (PMC12561962; doi:10.1371/journal.pone.0335192)
Supplement: S1 File — (DOCX) [file pone.0335192.s001.docx]

**Respondent Information Sheet**

**Research Title:**

Exploration of how early-career health professionals attempt to practise interprofessional collaboration in diabetes care in primary care setting.

**Introduction:**

You are invited to participate in a research titled as above. Before participating in this study, it is important that you take some time to read and understand the information in this information sheet.

**Purpose of Study:**

Diabetes mellitus (DM) care in Malaysia is still suboptimal and its management is complex. It is believed that DM care is not just involving multiprofessionals, but an interprofessional care is required which will be able to address the gaps of DM care management as it helps to improve patients care and outcomes. Hence, many medical and healthcare universities throughout Malaysia had started implementing interprofessional learning (IPL) into the curriculums to prepare health professionals graduate-to-be for interprofessional collaboration (IPC) in health workforce. However, when early-career health professionals (ECHPs) enter the primary care clinics, they are faced with several challenges in adapting and practising what they had learnt and experienced in university from IPL. Hence, the aim of this study is to explore and understand the process of how ECHPs attempt to practise IPC when managing DM in primary care setting and if IPL experiences during undergraduate training influence IPC practices.

**What would this involve?**

This is a qualitative approach research which will involve the participation of early-career health professionals who has worked in primary care clinics in DM care. This research will involve:

1. Obtaining consent from the participant to participate in the study.
2. Recording Background information on sociodemographic.
3. Setting an appointment with you for an in-depth one to one interview.
4. The expected in-depth one to one interview duration will be 45 minutes to 1 hour.
5. You and other participants will be either audio and/or video recorded in in-depth interviews.
6. Verbatim will be transcribed and analyse anonymously.

**Risk and benefits**

There are no anticipated risks as there are no interventions involved. This research will help to identify how IPC practices are being practised and what are the challenges and enablers of IPC which ECHPs encountered in the primary care setting. Furthermore, you will be given the chance to voice your opinion and thoughts on IPC practices in your own current work settings and management of DM. This would greatly help to promote IPC practices in each profession working in primary care clinics and for quality improvement in diabetes care management and its outcomes.

**Do I have to take part?**

- You may freely choose to withdraw from this study at anytime without reason and without repercussion.

**Data & Confidentiality:**

All the collected information from interview will be kept confidential and will only be reported as group data with no identifying information. All data will be kept in a secure location and only researchers directly involved with this research will have access to them.

**If I have any questions, whom can I ask at any time of the study?**

You may contact the following:

Associate Professor Dr Azimatun Noor Aizuddin

Department of Public Health Medicine, Faculty of Medicine, UKM

Email: [azimatunnoor@ppukm.ukm.edu.my](mailto:azimatunnoor@ppukm.ukm.edu.my)

Ms. Num Sze Fang (P102203)

PhD Candidate, Department of Public Health Medicine, Faculty of Medicine, UKM

Email: [p102203@siswa.ukm.edu.my](mailto:p102203@siswa.ukm.edu.my)

Professor Dr Mohd Shahrir Mohamed Said

Department of Medicine, Faculty of Medicine, UKM

Email: [shahrir@ppukm.ukm.edu.my](mailto:shahrir@ppukm.ukm.edu.my)
