# Supplementary material for: Navigating interprofessional collaboration in diabetes care: A qualitative study of early-career health professionals in malaysian primary care clinics
Source: PLoS One. 2025 Oct 28;20(10):e0335192. doi: 10.1371/journal.pone.0335192 (PMC12561962; doi:10.1371/journal.pone.0335192)
Supplement: S2 File — (DOCX) [file pone.0335192.s002.docx]

**INFORMED CONSENT FORM**

**Research Title: Exploration of how early-career health professionals attempt to practise interprofessional collaboration in diabetes care in primary care setting.**

Researcher’s Name:

1. Associate Professor Dr Azimatun Noor Aizuddin
2. Ms. Num Sze Fang (P102203)
3. Prof Dr Mohd Shahrir Mohamed Said

I, …………………………………………, IC No : ………………….........

- have read the information in the Respondent Information Sheet **including information regarding the risk in this study**
- have been given time to think about it and all of my questions have been answered to my satisfaction.
- understand that I may freely choose to withdraw from this study at anytime without reason and without repercussion
- understand I will be contacted when further information are required
- understand I will be video and/or voice recorded
- understand that my anonymity will be ensured in the write-up.

I voluntarily agree to be part of this research study, to follow the study procedures, and to provide necessary information to this research as requested.

………………………………. …………………..

(Signature) (Date)
