# Supplementary material for: Navigating interprofessional collaboration in diabetes care: A qualitative study of early-career health professionals in malaysian primary care clinics
Source: PLoS One. 2025 Oct 28;20(10):e0335192. doi: 10.1371/journal.pone.0335192 (PMC12561962; doi:10.1371/journal.pone.0335192)
Supplement: S3 File — (DOCX) [file pone.0335192.s003.docx]

**Supporting information**

S1 Interview topic guide

| **How Fresh Graduates Navigate Diabetes Care Through Interprofessional Collaboration In The Primary Care Setting? A Qualitative Study In Malaysia – Interview topic guide**  **Questions**   1. What do you understand about interprofessional collaboration? 2. Have you ever experienced interprofessional collaboration during practices at your current work? How was your experience? Can you give a few examples: Describe in terms of what happened, what your thoughts were and how did you feel about it? 3. Can you describe the practice of diabetes patients’ management in your clinic? 4. How would you describe your collaboration (if any) with the pharmacists, doctors, nurses and dietitian(s) you regularly work with in the diabetes care of your patients? 5. Has the collaboration changed any of the decisions you made, on your patient care? How so? Do they give input? 6. Would you be able to put forward what you think is appropriate about team care into practice? If yes, what makes that possible? If no, what makes that not possible, give some successful and unsuccessful examples 7. When you disagree with their opinion or recommendation, how did you manage the disagreement? 8. Is there anything else you would like to add on, or might we have missed? |
| --- |
